# **Ad**olescent type 1 **D**iabetes cardio-renal **I**ntervention **T**rial (**AdDIT**)

**DOI:** 10.1186/1471-2431-9-79

**Published:** 2009-12-17

**Authors:** The Adolescent type 1 Diabetes cardio-renal Intervention Trial Research Group

**Affiliations:** 1 Department of Paediatrics, University of Cambridge, Level 8 Box 116, Addenbrooke's Hospital, Hills Road, Cambridge, CB2 0QQ, UK

## Abstract

**Background:**

The prognosis for young people diagnosed with diabetes during childhood remains poor and this is mainly related to the long-term risk of developing vascular complications.

Microalbuminuria identifies subjects at risk for diabetic nephropathy (DN) and cardiovascular disease (CVD). It is often detected in adolescence but is rarely treated before the age of 18 years, as at the end of puberty albumin excretion may decline and in some subjects will return into the normal range. However, evidence indicates that subjects with both transient and persistent microalbuminuria have experienced renal damage during puberty and thus reno-protection to prevent long-term complications is warranted. In adults with diabetes and microalbuminuria, the use of angiotensin converting enzyme inhibitors (ACEI) and Statins is increasing, and in order to determine whether these agents are of value in the adolescent population a large randomized controlled clinical trial is needed.

**Methods/Design:**

The Adolescent type 1 Diabetes cardio-renal Intervention Trial (AdDIT) is a multi-center, randomized, double-blind, placebo-controlled trial of ACEI and Statin therapy in adolescents with type 1 diabetes. 500 high-risk adolescents, defined on the basis of their albumin excretion, are randomized to receive either ACEI (Quinapril) or Statins (Atorvastatin) or combination therapy or placebo for 3-4 years. There will also be a parallel open observational study, based on the follow-up of 400 low-risk non-randomized adolescents. The major endpoint of the study is the change in albumin excretion; secondary endpoints include markers of CVD, renal function, retinopathy, quality of life combined with assessment of compliance and potential health economic benefits.

**Discussion:**

AdDIT will provide important data on the potential renal and cardiovascular protective effects of ACEI and Statins in high-risk adolescents. Long-term follow-up of the randomized subjects will provide direct evidence of disease outcomes, in addition to the data on early surrogate measures of DN and CVD. Follow-up of non-randomized low-risk subjects will determine the potential impact of intervention on DN and CVD. AdDIT will help to determine whether, in addition to encouraging young people to achieve good glycaemic control, pharmacological cardio-renal protection should also be implemented.

**EudraCT Number:**

2007-001039-72

**Trial Registration Number:**

ISRCTN91419926

## Background

### Prognosis and complications of Type 1 Diabetes

The prognosis for childhood-onset type 1 diabetes (T1D) remains generally poor [1,2] and although life expectancy has increased by several years, reflecting increased longevity in the general population, the number of life years lost has remained unchanged over the last four decades and is about 17 years for a child diagnosed at the age of 10 years [3]. A recent study from Norway indicated that childhood-onset T1D is associated with a four-fold increase in the overall standardized mortality rate (SMR) [4], reflecting similar data from the USA [3]. By the age of 20 to 39 years the SMR for coronary heart disease in the British Diabetic Association Cohort of 23,000 patients diagnosed aged less than 30 years was increased ten-fold for men and forty-fold for women [2]. In the second decade after diagnosis diabetic nephropathy (DN) accounts for around 60% of deaths, whereas by the third decade cardiovascular disease (CVD) accounts for two thirds of all deaths [5]. However patients with nephropathy have approximately a forty-fold increased mortality from CVD [6]. The morbidity and mortality in childhood-onset diabetes is overwhelmingly associated with the development of long-term microvascular and macrovascular complications. Although complications are rarely seen during childhood, there is evidence that their pathogenesis begins soon after diagnosis and accelerates during puberty [7,8]. Thus, adolescence may be a critical period for lifetime risk of complications in childhood onset T1D.

### Seeds of future complications in adolescence

Glycated hemoglobin levels (HbA1c) during puberty are invariably higher than levels recommended for prevention of complications. In the Diabetes UK National Audit the mean HbA1c in those aged under 16 years was 8.9% and only 72% had an HbA1c less than 9.5% [9]. In the Diabetes Control and Complications Trial (DCCT), although adolescents showed the same benefits from intensified therapy as adults, HbA1c levels were generally 1% higher and excess weight gain and hypoglycaemia were more frequent in the adolescents [10,11].

It is during puberty that the first signs of complications become evident and microalbuminuria (MA), an early risk marker for DN and CVD [12,13] may be found in 12-16% of adolescents [13-17]. This has been associated with renal pathology indicative of early nephropathy [18]. The relationship between puberty and MA is only partly explained by poor glycaemic control and there is evidence that puberty itself may be an independent risk factor [13,19]. The development of MA is associated with hyperlipidemia [20,21], elevation of arterial blood pressure [22], decline in renal function [23] and retinal changes [24]. It has been suggested that MA represents the first evidence of a generalized endotheliopathy [25]. Flow mediated dilation (FMD), an established marker of endothelial function, may be abnormal in adolescents with T1D [26] and carotid artery intima-media thickness (cIMT), a marker of early atherosclerosis and a strong predictor of future vascular events [27], has been found to be increased in adolescents with T1D [26,28-32]. Markers of sub-clinical atherosclerosis, including cIMT, have been linked to the development of MA in the general adult population [33]. Markers of endothelial dysfunction and arterial stiffness have also been associated with MA in diabetic populations [34,35]. Thus rapid growth and poor glycaemic control during adolescence both contribute to the future complications risk associated with MA.

### Intervention in adolescents to prevent future complications

It has been argued that early intervention in adolescents with MA is unnecessary as it may revert to normal at the end of puberty in 50% of patients [36-40] and only around 15% will progress to clinical proteinuria. Recent data from the Oxford Regional Prospective Study (ORPS), which has been following an incident cohort of around 500 children since 1986 [13,41], indicate that MA is transient in 39% and with continued follow up, 48% of subjects have persistent MA and 13% have intermittent MA. 4% of the ORPS cohort has developed clinical proteinuria and this is related to both persistent (hazard ratio (HR): 27.7) and intermittent MA (HR 8.8) [41]. The cumulative probability of developing clinical proteinuria was 13.9% after 18 years diabetes duration in this population, diagnosed at 9.5 years [41] compared with a rate of 14.6% in the only adult incident cohort after a similar duration of diabetes [42]. Risk for MA can be identified by greater rates of albumin excretion, although still within the normal range, as early as one year from diagnosis [43] and we have recently shown that albumin excretion in the highest tertile after adjustment for age, gender, duration of disease and age of diagnosis, at age 11-15 years, predicts 85% of subjects who go on to develop MA and all of the subjects who develop clinical proteinuria [44]. Therefore early abnormalities in albumin excretion during adolescence are predictive of complications risk and early intervention in high-risk adolescents could lead to long-term improvements in prognosis.

### Previous interventions with ACEI and Statins

T1D adults with both intermittent and persistent MA are increasingly being treated with angiotensin converting enzyme inhibitors (ACEI) or angiotensin receptor blockers (ARB), as these drugs have been shown to reduce risk of progression of MA to clinical proteinuria and may also be effective in reducing mortality from CVD [45]. Screening for MA is routinely recommended during adolescence [46,47], yet recommendations for treatment are not consistent. Four small studies have confirmed the likely efficacy of ACEI in adolescents with MA, but there have been no formal randomized controlled trials (RCTs) [48-51]. In a review of 12 adult trials of ACEI, reductions in albumin excretion rates of 50.5% (95% CI: 29.2-65.5) [52] were similar to those observed in the adolescent studies: 41% [48] and 55-60% [49]. In the study reported by Rudberg [53], reductions in albumin excretion were associated with improvement in renal biopsy changes in young patients with T1D. A beneficial effect of ACEIs treatment, has also been demonstrated for diabetic retinopathy [54]. The EURODIAB Controlled Trial of Lisinopril in T1D showed a significant effect of lisinopril in reducing by around 50% the progression of retinopathy in normotensive and normo- or microalbuminuric patients [54]. In the recent Renin-Angiotensin System Study, treatment with either enalapril or losartan reduced progression of retinopathy by 65-70% [55].

Hyperlipidemia is commonly reported in adolescents with T1D [20,21] but there have been no studies of statin therapy in this population. Treatment with HMG-CoA reductase inhibitors (Statins) is becoming increasingly common in adults as they reduce major vascular event rates by about one third in T1D subjects aged > 40 years [56]. The National Institute for Health and Clinical Excellence guidelines recommend statin treatment for T1D adults with MA or two or more features of the metabolic syndrome and the recent Joint British Societies' Guidelines [57] recommend statin treatment in adults with persistent MA. Management depends on extrapolating from the results of trials conducted in adults with diabetes [58] and short-term trials of up to two years duration conducted in children with familial hypercholesterolemia (FH) that show treatment to be efficacious and safe [59,60]. Longer-term trials in children and adolescents with T1D are therefore urgently needed to address clinical uncertainty [61].

Clinical trials may need to use surrogate endpoints and non-invasive vascular assessment techniques have demonstrated abnormalities in T1D in early childhood [26,28-32,62]. In particular, cIMT has been shown to be significantly greater in children with T1D than in age-matched controls [26] and not dissimilar to that observed in heterozygote subjects with FH [63]. Other measures of CVD risk and endothelial dysfunction, such as FMD, may precede changes in cIMT within the first decade of T1D in children [26] and other measures such as endothelial pulse amplitude tonometry (EndoPAT) may also distinguish T1D from healthy subjects [64-66]. Arterial stiffness as assessed by pulse wave velocity (PWV) may be an additional marker of CVD, which has been shown to be abnormal in T1D patients as young as 10 years [67]. Statin treatment would be expected to slow the progression or result in regression of cIMT, and a two-year RCT of pravastatin in 214 children with heterozygous FH found active treatment to result in a significant difference in the mean change in cIMT between the two groups [68]. In addition to reducing the risk of CVD, statin therapy may also reduce the risk of nephropathy. Low density lipoprotein (LDL) cholesterol, and small dense LDL in particular, produce proliferative and biosynthetic responses in glomerular cells that occur in the early stages of DN [69]. Mesangial cells can take up both native and modified LDL and respond in vitro by proliferation, production of chemo-attractants for monocytes and macrophages, and alterations to matrix turnover leading to net matrix expansion [70]. Furthermore, diabetes induced mesangial expansion has been shown in animal models to be reduced in a dose dependent manner by statins [71]. Statin treatment is associated with a significant reduction in mean serum creatinine concentration [72] and a recent trial of subjects with diabetes reported a reduction of 11% in patients randomized to atorvastatin for three years whilst patients randomized to standard care showed a 5% increase in creatinine [73]. These findings suggest that statin therapy in T1D may reduce the risk of both DN and CVD. Dyslipidemia has also been implicated in the pathogenesis of retinopathy and consequently statin treatment may be beneficial in limiting this complication [74,75]. Combination therapy with a statin and an ACEI for patients at high risk of development of MA may therefore represent the most effective strategy for reducing future morbidity and mortality from DN and CVD in patients with childhood-onset T1D. Thus both ACEI and Statin therapy could provide cardio-renal protection in high-risk subjects during adolescence and we plan to explore this hypothesis through a randomized controlled trial.

## Aim Of The Study

To carry out a multi-center, randomized, double-blind, placebo-controlled trial of ACEI and Statin therapy in adolescents with T1D. 500 adolescents at high risk for DN and CVD will be randomized to receive either ACEI or Statins or combination therapy or placebo for three to four years (**Study 1**). There will also be a parallel open observational study, based on the follow-up of 400 low-risk non-randomized adolescents (**Study 2**).

## 1. Randomised Controlled Clinical Trial (Study 1)

### Study Objectives

#### Primary

To determine whether intervention with ACEI, Statins, or combination therapy when compared with placebo, in a 2 × 2 factorial design over three to four years, will: 1) reduce albumin excretion as assessed by six monthly measurement of albumin/creatinine ratio (ACR) in 3 early morning urines; 2) reduce the incidence of MA (ACR >3.5 mg/mmol (males) or >4 mg/mmol (females) in 2 out of 3 urines) at the end of the study period; 3) reduce the incidence of MA during the six month run-out period following the completion of intervention phase.

#### Secondary

To determine the effects of the intervention on: 1) changes in cIMT; endothelial function and arterial stiffness; 2) changes in arterial blood pressure (BP), blood lipids and lipoproteins; 3) changes in glomerular filtration rate (GFR) as assessed by plasma symmetric dimethylarginine (SDMA), creatinine and cystatin C levels; 4) changes in CVD risk markers: high-sensitivity C-reactive protein (hsCRP) and asymmetric dimethylarginine (ADMA); 5) incidence of retinopathy and subclinical changes in the retinal microvasculature; 6) quality of life, risk benefit, compliance and health economic assessment; 7) long-term outcomes with regard to incidence of DN and CVD.

### Study endpoints

The primary endpoint is defined as the area under the curve over time of log ACR per year, with standardization for gender, age and duration of diabetes.

The secondary endpoints are: 1) changes in cIMT, FMD, EndoPAT and PWV between baseline and the end of intervention period; 2) changes in arterial BP, lipids and other lipoproteins, CVD risk markers (hsCRP and ADMA), assessed every 6 months during the intervention period; 3) changes in measures of GFR (plasma SDMA, creatinine and cystatin C) assessed every 6 months during the intervention period; 4) changes in retinopathy scores and retinal microvascular structure (arteriolar or venular dilation, vascular fractal dimension, branching and tortuosity), assessed annually; 5) changes in quality of life and health economics.

### Methods/Design

#### Design of the RCT

The study is a multicenter randomized trial involving centers in the UK, Australia and Canada *(see additional file *1).

Subjects will be recruited from a pre-screened population of ~3,000 young people with T1D aged 11 to 16 years based on assessment of risk for future CVD and DN. Subjects deemed to be at high risk, based on their albumin excretion rate, will be randomized to a 2 × 2 factorial design contrasting the effects of ACEI, statins, or combination therapy to placebo over a three to four year treatment period. Minimization of variation between study arms in albumin excretion rate, gender, age, diabetes duration, HbA1c, total cholesterol and center location will be undertaken at randomization.

Analysis of the primary endpoint, change in albumin excretion, will be undertaken on an 'intention to treat' basis. Secondary analyses will be undertaken on the basis of 'as treated' allowing for variance in compliance and allowing for subjects who show substantial changes in HbA1c levels. Additional analyses will be undertaken to assess changes in the secondary objectives and to assess the overall effect of the intervention on quality of life and health economics.

#### Study population for the RCT

##### • Definition of high-risk subjects

Subjects with T1D within the age range 11-16 years are screened in the UK, Australia and Canada to determine the ACR in two sets of 3 early morning urines. The definition of a high-risk subject is based on previous data derived from the ORPS and Nephropathy Family Study (NFS) cohorts [41,76].

Preliminary data from the ORPS cohort (1,697 sets of urines from 479 subjects) indicated that the log standardized ACR based on the residuals from a regression model of log ACR adjusted for age, gender, age at diagnosis and duration of diabetes at 11-15 years was highly predictive of risk for the development of MA [44]. The average residual across visits provided a robust estimate of standardized ACR and a value above the cut-off point of 1.2 defines the high-risk upper tertile. A validation of the applicability of the ORPS ACR cut-off point and regression coefficients to another cohort was conducted in 690 subjects aged 11-15 years with ACR measures from two sets of 3 urines in the NFS. 27.5% had standardized ACR values in the top tertile; 93% of those selected using the ORPS coefficients and cut-off point would also have been selected with revised coefficients. This indicates good reproducibility, in a context where the mean HbA1c was 1% lower in the NFS when compared with the ORPS cohort.

During screening for AdDIT, two mean ACR measures, each based on three early morning samples, will have been provided by a subject. These will be averaged on the log ACR scale and the subject's average residual will be calculated using gender, age and duration and the coefficients from the ORPS linear regression model. If the subject's residual lies above log 1.2 the patient will lie in the upper tertile indicating high risk and eligibility for the trial.

*Inclusion criteria*

1) Age 11 to 16 years; 2) T1D diagnosed for more than 1 year or C-peptide negative; 3) Centralized assessment of ACR based on six early morning urines deemed to be in upper tertile for risk after adjustment for age, gender, age at diagnosis and duration of disease.

*Exclusion Criteria*

the presence of any of the following will prevent patient inclusion: 1) Non T1D, i.e. type 2 diabetes, insulin dependent diabetes related to monogenic disease, secondary diabetes; 2) ACR based on six early morning urines deemed to be at low risk for subsequent development of CVD or DN; 3) Pregnancy or unwillingness to comply with contraceptive advice and regular testing throughout trial; 4) Breast feeding; 5) Severe hyperlipidaemia and family history data to support diagnosis of familial hypercholesterolaemia; 6) Established hypertension unrelated to DN; 7) Prior exposure to the investigational products; 8) Unwillingness/inability to comply with the study protocol; 9) Other co-morbidities considered unsuitable by the investigator (excluding treated hypothyroidism and celiac disease); 10) Proliferative retinopathy.

##### • Sample size determination and power calculations

*Number of Subjects required*

The primary power calculation estimates that 400 subjects will need to be analyzed and we aim to recruit 500 to allow for dropouts and non-compliance.

*Power calculations based on the primary endpoint*

A 30% reduction in ACR is considered a worthwhile and plausible effect size to detect in this population and detection of a 25% reduction would be an advantage. With the 2 × 2 factorial design we plan for follow-up of 100 patients in each of the four arms: Placebo (P), ACEI (A), Statin (S), combination of ACEI and Statin (C). This sample size is informed by the ORPS cohort study (477 participants in the correct age range of recruitment with a mean of 3.5 observations over time) [13]. Within the tertile with the highest ACR standardized for gender, age and duration (defined as standardized ACR > 1.2), the standard deviation (SD) of standardized log to base 10 of ACR was 0.38 (mean 0.28) based on analyzing the final value alone, whereas the SD of the average standardized log ACR repeated over time, defined as area under curve per year, was 0.28 offering a reduction in detectable effect size from 30% to 25%. The primary endpoint is therefore defined as area under curve of log standardized ACR, with standardization for gender, age and duration of disease. The chosen sample size and endpoint provide 93% power to detect a 25% reduction in ACR attributable to the factorial main effect of ACEI (A and A+S arms [n = 200 total] versus P and S arms [n = 200 total]), and of Statin (S and S+A arms [n = 200 total] versus P and P+A [n = 200 total]). Additionally there is 82% power to detect a 30% reduction in ACR, in the following single-arm comparisons: A [n = 100] versus P [n = 100]; S [n = 100] versus P [n = 100]; and S+A [n = 100] versus P [n = 100].

*Power calculations for secondary endpoints*

For secondary outcomes, a 1% level of significance will be applied. For continuous outcomes, the sample size allows detection of small to moderate effect sizes; there will be 82% power in single arm comparisons to detect effect sizes of 0.5 SD between arms, and 93% power for factorial main effects of size 0.4 SD. For the secondary outcome cIMT, a pooled estimate across two studies of the SD of change from baseline is 0.044 [68]. There is 93% power to detect a 0.018 mm difference for ACEI and Statin factorial main effects, and 82% power to detect a 0.022 mm difference for single-arm comparisons.

For the secondary outcome FMD, an estimate across recent studies of the SD of change from baseline is 2.5. 180 subjects will give 80% power to detect a 1.5% difference in FMD between baseline and final assessment at a 5% level of significance.

For the secondary outcome PWV, an estimate across recent studies of the SD of change from baseline is 1.5. 200 subjects will give 80% power to detect a 0.85 m/sec difference in PWV between baseline and final assessment at a 5% level of significance.

For the secondary outcomes retinopathy and subclinical changes in the retinal microvasculature, the sample size will give greater than 90% power to detect a 25% difference in retinopathy prevalence and to detect a minimum of 3% change in retinal vascular parameters.

For binary outcomes, the sample size allows detection of moderate to large difference in proportion effect sizes, when the proportion is the mid-range of 0.2 to 0.8; so that there is 93% power to detect factorial main effects of a 50% relative reduction for proportions 0.4 versus 0.2, and the corresponding relative reduction detectable for the single-arm comparisons is 58%. For the secondary outcome of MA over the two measurements at age 18, the detectable reduction for factorial main effects is prohibitively large, 75%, due to a necessarily low prevalence in the treated arm, and therefore the more sensitive continuous ACR outcome will be the primary indicator of MA.

#### Study procedures (Figure 1)

**Figure 1:**
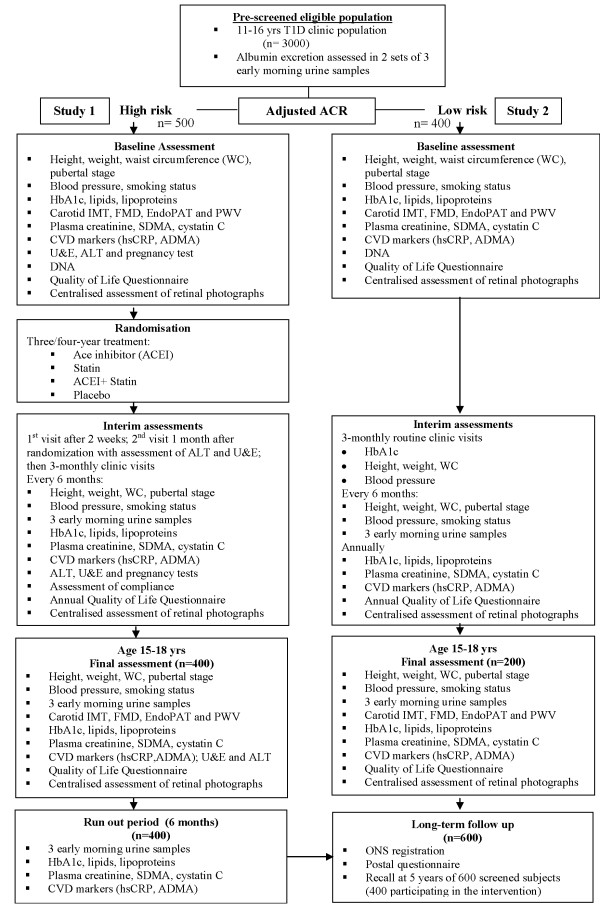
**Combined study flow-chart**.

##### Recruitment

Eligible subjects and their families will be approached by the research nurse and the local principal investigator (PI) and they will be provided with a verbal explanation of the study and written information sheets. Once they have been given sufficient time to consider their participation in the trial, the parents will be asked to provide written informed consent and children to provide evidence of their assent to the study procedures.

##### Baseline Assessment

Once consent has been obtained, baseline assessments will be undertaken within 3 months of recruitment. These will include measurement of height, weight, waist circumference (WC), arterial BP and assessment of pubertal stage. Blood samples will be taken for measurement of HbA1c, total cholesterol, triglycerides, HDL-cholesterol, LDL-cholesterol, lipoproteins, SDMA, creatinine, cystatin C, CVD markers (hsCRP and ADMA) and DNA extraction. All subjects will also have alanine aminotranferase (ALT) and urea and electrolytes (U&E) assessed locally and post-menarchal sexually active girls will have pregnancy tests. All subjects will be asked to attend designated centers for cIMT assessment and, where facilities allow, also FMD, EndoPAT and PWV. They will be asked to complete a simple quality of life questionnaire and a short questionnaire for Health Economic analysis. Anonymised digital copies of the most recent retinal photographs, performed as part of the routine annual screening, will be also collected for centralized assessments.

##### Randomization

Eligible subjects who have provided informed consent and have completed baseline assessments will be randomized by the PI using a secure internet-based service http://www.sealedenvelope.com. This provides randomization with minimization [77]. Subjects will be allocated to one of four treatment regimens after minimizing differences between arms on the following baseline characteristics: HbA1c (<7.5, 7.5-8.5, > 8.5%), log mean standardized ACR (>1.2 to <1.7, >1.7), gender, age (11-13, >13 years), duration of disease (<5 years, >5 years), total cholesterol (≥ 4.46 or < 4.46 mmol/l).

##### Initiation of therapy

Following confirmation of patients' eligibility for the trial they will be randomized, and intervention will commence. Subjects will be instructed by the research nurse/PI about the administration and the storage of the investigational medical products (IMPs). Furthermore, the patients and their parents will be informed about possible side effects related to the drug and will be instructed to report any adverse events during treatment. Contact during the first four weeks of treatment will be maintained weekly by the research nurse, through visits or by telephone at pre-arranged times.

##### Treatment period assessment

Subjects will be seen two weeks after commencing treatment for assessment of compliance, adverse events and adjustment of the dose of Quinapril/Placebo. Subjects will be seen again after one month, when toxicology tests will be also performed (ALT and U&E). Every three months subjects will undergo a routine clinical assessment with local measurement of HbA1c. Six-monthly assessment will include: three consecutive early morning urines for ACR, height, weight, waist circumference, pubertal stage, BP, smoking status, HbA1c, blood lipids and lipoproteins, plasma SDMA, creatinine, cystatin C and CVD biomarkers, toxicology (ALT, U&E) and pregnancy tests. Compliance will be assessed by return of study drugs and drug bottles with electronic track caps. Quality of life and health economics will be assessed annually with a questionnaire. At each annual visit, subjects will be asked whether they have undergone routine annual retinal screening and anonymised digital copies of retinal photographs will be collected for centralized assessment.

##### Final assessment

The final assessment after 3-4 years of the intervention will be identical to the initial assessment and will include measurement of cIMT and, where facilities allow, FMD, EndoPAT and PWV. Anonymised digital copies of the most recent retinal photographs, performed as part of routine screening, will be collected for centralized assessment.

##### Run-out assessments

At the end of the intervention period and following the final study assessment, all study drugs will be withdrawn and subjects will be followed for a 6-month run-out period. At the end of this period subjects will be asked to provide a final blood sample and three early morning urines for assessment of ACR and MA status and measurement of lipids, lipoproteins, CVD risk markers, SDMA, creatinine and cystatin C. These data will be used to inform physicians as to the need for re-starting IMP according to local prescribing guidelines in subjects aged 15-19 years.

##### Long-term follow-up

Consent is obtained in the initial consent procedures for possible long-term surveillance through postal questionnaires and possible recall 5 and 10 years after study initiation. In the UK registration with the Office for National Statistics (ONS) may also be sought.

#### Investigational Medical Products (IMPs)

The active drugs (Atorvastatin and Quinapril) will be manufactured by Pfizer, along with appropriate placebos with adherence to Good Manufacturing Practices. Atorvastatin will be provided in a single dose of 10 mg daily for oral administration with an identically labeled placebo (Study drug A). Quinapril will be available at 2 doses: 5 mg and 10 mg with appropriately matched placebo (Study drug Q). Subjects will be started on 5 mg and reviewed after two weeks; if there have been no adverse reactions in this time, the dose will be increased to 10 mg daily, otherwise it will maintained at 5 mg. Any subjects experiencing apparent side effects (postural hypotension or persistent cough) will have the option of changing to the lower dose at any point during the study.

Subjects will be instructed to take two tablets per day; Pharmacists will be instructed to dispense the blinded product according to the schedule and advised that dose of Study drug Q may need to be reduced. All drugs will be given orally as tablets. No subject will be exposed to more than 10 mg of Quinapril or 10 mg of Atorvastatin. Subjects will be treated with the IMPs for a minimum of three years and up to four years depending on age at recruitment.

Both ACEI and Statins are licensed for use in children and adolescents in the UK, Australia and Canada. However the product licenses are related to the treatment of hypertension in non-diabetic subjects and the treatment of hyperlipidaemia in subjects with familial hypercholesterolaemia. To facilitate the use of the drugs in children with T1D an appropriate Clinical Trial Authorization has been obtained from regulatory authorities in the UK, Canada and Australia.

The drugs and placebo will be delivered to Catalent Pharma Solutions, the contracted distribution company that will be responsible for packaging, labeling and distribution of the IMPs to the designated pharmacies at the study sites. Electronic pill bottle monitoring caps supplied by AARDEX (Zug, Switzerland) will be used. These track caps incorporate a micro-electronic circuit, which records each opening and closing in order to track compliance.

Central pharmacies will dispense the IMPs to the study subjects through the research nurses employed by the local PIs. The nurses will then instruct the patients about the proper storage and use of the drugs, including the return of all unused tablets. IMPs will be dispensed according to local requirements as stated in Standard Operating Procedures (SOPs) and taking account of local regulations.

Common adverse drug reactions have previously been reported for Atorvastatin [78] and Quinapril [79]. For the purposes of adverse event reporting during study visits, particular attention will be paid to possible hyperkalemia, persistent dry cough, hypotension, GFR decline, ALT levels for liver function and muscular pain/rhabdomyolysis. Both Quinapril and Atorvastatin are contra-indicated during pregnancy because of concern about teratogenecity and because they may interfere with early fetal growth and result in stillbirth. See additional file 2 for more information.

#### Blinding and Emergency unblinding

The subject, local investigator and all study and project management staff will be blinded as to treatment allocation. Subjects will be randomized using the web-based system and the Cambridge coordinating center will receive notification of randomization within 24 hours. The randomization website http://www.sealedenvelope.com provides a facility for emergency unblinding of drug allocation. Each PI will be provided with a password which will allow access to their patients' treatment allocation. All emergency unbinding will be at the discretion of the local investigator, when clinically indicated for the safety of the patient. Unblinding will also be possible through the pharmacy. Investigators should refer to SOPs for the unblinding procedure. All unblindings are automatically notified by email to the study-coordinating center in Cambridge to prevent abuse of the system and to ensure rapid notification to the data monitoring and ethics committee (DMEC) of potential serious adverse events (SAEs).

#### Compliance

Subject compliance will be assessed at each 3-monthly study visit, when all unused study drug will be returned and will be recorded in the CRFs by the research nurses. AARDEX electronic pill bottle track caps, which permit checks of the number of times the pill container has been opened between study visits [80,81], will also be used. The track cap data will be available locally through software provided by AARDEX and can be used by the study nurses to encourage compliance and where appropriate introduce interventions to improve compliance. We will also make regular contact with study subjects through newsletters identifying the value of continued involvement in the study protocol.

#### Withdrawal of study drugs

With exception of pregnancy, if it is deemed necessary to withdraw a patient from one of the study drugs this will not preclude their continuing participation in the study. Subjects withdrawn from either of the study IMPs will be encouraged to remain in the trial.

#### Rescue medications

Patients developing clinical hypertension will be treated at the discretion of the local investigator. Treatment is initially expected to be a diuretic, followed by the use of a calcium channel blockers (or similar agent). Very high cholesterol levels, perhaps related to familial hypercholesterolaemia rather than T1D will be identified at baseline as an exclusion criterion. However, if at any time during the intervention period LDL cholesterol levels are found to be above 4.6 mmol/l and/or triglyceride levels are above 9 mmol/l, the central laboratory will notify the site. A first attempt to reduce lipids levels will be made by improving the patient's glycaemic control and dietary intervention; a second lipid measurement will be performed within four weeks. If the second value is still above the threshold, the case will be notified to the DMEC for consideration of withdrawal from the study. If at any time during the study, the ACR is in the macroalbuminuric range (ACR>35 mg/mmol in males and >47 mg/mmol in females [13]), these cases will be notified to the DMEC for consideration of withdrawal from the study

## 2. Study 2: Follow-Up Of Non-Randomised Low Risk Subjects

### Study objectives

The primary objective of **Study 2 **is to determine the relationship between albumin excretion and cIMT both at baseline and after 3-4 years in subjects deemed to be at low risk on the basis of urinary albumin excretion when aged 11-16 years.

The secondary objective is to determine the relationship between albumin excretion and changes between baseline and 3-4 years in: 1) additional measures of subclinical atherosclerosis, such as endothelial function and arterial stiffness; 2) CVD risk markers: hsCRP and ADMA; 3) arterial BP, blood lipids and lipoproteins; 4) GFR as assessed by SDMA, creatinine and cystatin C levels; 5) long-term outcomes with regard to incidence of DN and CVD.

### Study endpoints

The primary endpoint is the relationship between ACR and cIMT and how this changes during puberty.

The secondary endpoints are: 1) changes in FMD, EndoPAT, PWV between baseline and the end of the study; 2) changes in arterial BP, lipids and other lipoproteins, CVD risk markers (hsCRP and ADMA), assessed annually; 3) changes in measures of GFR (plasma SDMA, creatinine and cystatin C), assessed annually; 4) changes in retinopathy scores and in the retinal microvasculature (arteriolar or venular dilation, vascular fractal dimension, branching and tortuosity), assessed annually.

### Method/Design

#### Study design

This will be an open observational study of low-risk subjects in the UK. In the UK, 400 subjects not recruited into the interventional study (from the medium and low tertiles of ACR) will be assessed at baseline with measurement of cIMT (and FMD, EndoPAT and PWV where facilities allow), BP, lipids, HbA1c, and CVD markers and renal function. Copies of the most recent digitalized retinal photographs, performed as part of the annual routine screening, will also be collected and retained for centralized assessments. These subjects, together with 200 high risk individuals in the UK from the RCT will form the cohort of 600 subjects to enable the study of the relationship between markers of sub-clinical atherosclerosis (cIMT, endothelial function and arterial stiffness), and albumin excretion and its modification by cardiovascular risk factors, renal function and diabetes control. 200 of the low risk subjects will be re-assessed annually and 3-4 years after initial screening. The findings will be compared to treated subjects from the interventional trial including 100 high risk subjects randomized to placebo arm. The aim is to provide additional data concerning the natural history of renal, cardiac and retinal measures in subjects deemed to be at different levels of risk.

#### Study population

##### • Definition of low-risk subjects

As in the RCT, during the screening phase of the study two ACR measures, each based on three early morning urine samples, are to be provided by a subject. These will be averaged on the log ACR scale and the subject's average residual will be calculated using gender, age, age at diagnosis and duration of diabetes, as well as the coefficients from the ORPS linear regression model. If the subject's residual lies below log 1.2 the patient will lie in the medium or lower tertile indicating low risk and eligibility for the non-randomized study.

• Inclusion and exclusion criteria

*Inclusion criteria*

1) Age 11 to 16 years; 2) T1D diagnosed for more than 1 year or C-peptide negative; 3) Centralized assessment of ACR based on six early morning urines deemed to be in middle or lower tertiles for risk after adjustment for age, gender, age at diagnosis and duration of disease.

*Exclusion Criteria*

The presence of any of the following will preclude patient participation: 1) Non T1D, i.e. type 2 diabetes, insulin dependent diabetes related to monogenic disease, secondary diabetes; 2) ACR based on six early morning urines deemed to be at high risk for subsequent development of CVD or DN; 3) Severe hyperlipidaemia and family history data to support diagnosis of familial hypercholesterolaemia; 4) Established hypertension unrelated to DN; 5) Prior exposure to the investigational products, statins and ACEI; 6) Other co-morbidities considered unsuitable by the investigator (excluding treated hypothyroidism and celiac disease); 7) Proliferative retinopathy.

##### • Sample size determination and power calculations

Based on a previous pediatric cohort of 45 patients with T1D with mean cIMT of 0.58 (SD 0.05), a sample size of 600 subjects at baseline (200 subjects evaluated per tertile of ACR) would allow mean cIMT in this population to be estimated precisely with 95% confidence interval of ± 0.004 mm and would provide 83% power to detect an unadjusted correlation of ≥ 0.12 between cIMT and ACR (α = 5%). This will allow sensitive detection of the association between ACR and cIMT after adjustment for potential confounders (such as age, gender, blood pressure, lipids, inflammation and HbA1c) using multiple linear regression.

For the analysis including data from baseline and at the end of the study, 300 subjects (100 high risk subjects from the RCT plus 100 low risk subjects from the middle and lower tertiles of ACR), the study will provide 80% power to detect correlation coefficients, such as between cIMT and ACR ≥ 0.16 (α = 5%). Assuming the same standard deviation of 0.044, analysis of the trend in cIMT across all three tertiles of ACR allows a range of ≥ 0.035 mm in mean cIMT to be detected across tertiles, and ≥ 0.022 mm between two tertiles. The analysis will include a regression model incorporating the 600 cIMT measures from subjects where cIMT was measured at both baseline and follow-up, to estimate the shape of progression of cIMT by gender and age over time and in relation to puberty and other potentially predictive characteristics.

For FMD, an estimate across recent studies of the SD of change from baseline is 2.5. 70 subjects will give >90% power to detect a 1% difference in FMD between baseline and final assessment at a 5% level of significance. For PWV, an estimate across recent studies of the SD of change from baseline is 1.5. 95 subjects will give 90% power to detect a 0.5 m/sec difference in PWV between baseline and final assessment at a 5% level of significance.

#### Study Procedures (Figure 1)

##### Recruitment

Eligible subjects and their families will be approached by the research nurse and the local PI and they will be provided with a verbal explanation of the study, and written information sheets. Once they have been given sufficient time to consider their participation in the trial, the parents will be asked to provide written informed consent and children to provide evidence of their assent to the study procedures.

##### Baseline assessment

Once consent has been obtained, baseline assessments will be undertaken within 3 months of recruitment. These assessments will include: cIMT (and FMD, EndoPAT and PWV where facilities allow), blood samples for lipids and lipoproteins, CVD risk markers, SDMA, creatinine, cystatin C, three early morning urines for ACR and collection of anonymised digital copies of the most recent retinal photographs, performed as part of the annual routine screening, for centralized assessments. These subjects will also be asked to complete annual questionnaires regarding quality of life and to provide data for economic assessments during the study period. It is anticipated that 200 of these subjects will agree to the assessment of ACR (3 urines), blood tests and cardiovascular assessments and to retain copies of their retinal photographs for centralized assessment at the end of the study period. This will provide additional data concerning the natural history of these markers of complications risk. These 200 subjects will be asked to consent to long-term follow up and re-assessment five years after the end of the study period.

##### Follow-up assessments

As routine, subjects will be seen quarterly in clinics with local assessment of HbA1c to facilitate appropriate medical care and where appropriate intensification of insulin therapy. Height, weight, waist circumference and arterial BP will also be assessed at each visit.

Every 6 months, three early morning urine samples will be collected for centralized analysis of the ACR. Pubertal stage and smoking status will also be assessed.

Annually, blood samples will be taken for lipids and lipoproteins, CVD risk markers, SDMA, creatinine, cystatin C and subjects will also be asked to fill in a questionnaire to provide control data for quality of life and health economics assessment of the intervention cohort. Anonymised digital copies of the most recent retinal photographs, performed as part of the annual routine screening, will be collected and retained for centralized assessment.

#### Long-term follow up of screened and intervention cohort (Study 1 and Study 2)

The primary and secondary outcomes of the study are robust, but they are surrogates for long-term DN and CVD risk. It is therefore essential that planned follow-up of the cohort is undertaken. In the UK all of the subjects, both randomized and non-randomized controls will be flagged by the UK Office for National Statistics (ONS) in order to track them through the UK National Health Service database and to permit tracing and outcome assessment by local consultants and to receive notifications of deaths. We will undertake similar tracking of subjects in Canada and Australia using national health numbers and diabetes registers.

In addition we plan formal re-assessment of all randomized (400) and non-randomized subjects (UK 200) five years after the end of the study, subject to successful completion of the study and securing further funding. This assessment would include cIMT (and FMD, EndoPAT and PWV where facilities allow) and markers of CVD risk combined with a comprehensive assessment of microvascular complications including urinary albumin excretion, GFR and retinal photographs. To facilitate this long-term follow-up we will endeavor to keep in touch with the cohort through annual newsletters.

## 3. Common Methods For Study 1 And Study 2

### Data management

At screening, all subjects are given a unique identifying number which will be translated into a barcode used for all subsequent correspondence, transfer of samples and data input to the centralized database. Study databases are developed "in house" and incorporate quality control (QC) checks to ensure accurate data entry data. Standardized CRFs have been developed for use in all centers. Results from the Central Laboratory for screened subjects will be available on a secure website in order to identify those eligible for recruitment and randomization.

Data from the CRFs will be entered onto a secure locally held database at each center with anonymised data regularly sent to the central database held at the Clinical Trials Unit in Cambridge. Off line checks will be run on data received to identify missing or inaccurate data and queries will be fed back to centers using existing SOPs. A random 10% of CRFs from each center will be double data entered by staff in Cambridge to provide an additional QA check.

### Laboratories

The WellChild laboratory at The Evelina Children's Hospital, London will undertake centralized assessments of all urinary screening samples, 6 monthly study urine samples and bloods for HbA1c, creatinine, SDMA, cystatin C, fasting lipids, lipoproteins, ADMA, and hsCRP. Urine albumin will be measured using laser immunonephelometry (Dade Behring) and for concentrations <2.1 mg/l by an enzyme linked immunoabsorbent assay (ELISA). Urine creatinine will be measured using a chromatographic stable isotope dilution electrospray mass spectrometry-mass spectrometry (MSMS) method. HbA1c % will be measured in filter paper blood spots, after endoproteinase Glu-C digestion, using an MSMS reference method measuring the -N-terminal hexapeptides of HbA1c and HbA0. GFR will be monitored using plasma creatinine measured by a NIST traceable MSMS method and estimated GFR (eGFR) calculated from the formula: eGFR (ml/min/1.73 m^2^) = 42*height (cm)/plasma creatinine (μmol/l). GFR will also be monitored using plasma SDMA, measured by MSMS, and cystatin C, measured by laser immunonephelometry (Dade Behring); both GFR markers are independent of body size [82-84].

CVD biomarkers will include fasting lipids and lipoproteins, measured using routinely available methods, hsCRP by laser immunonephelometry (Dade Behring), and ADMA by MSMS. Serum and plasma will be stored at -80°C for future analysis of any compelling biomarkers. Local site-specific laboratories will analyze toxicology data (U&E, ALT) and pregnancy tests and details of local methods, normal ranges, QC and QA will be obtained during site set up visits and confirmed during monitoring visits. 3-monthly HbA1c will be also assessed in local laboratories, using DCCT aligned methods.

### Cardiovascular assessments

cIMT will be assessed at baseline and the end of the study in designated centers in the UK, Australia and Canada, through collaborative agreements. Where facilities and training allow, centers will also provide endothelial function assessment (by FMD and EndoPAT) and arterial stiffness assessment (by PWV) at baseline and at the end of the study.

cIMT will be quoted as end-diastolic mean-mean and end-diastolic mean-max. The maximum IMT measurement of a segment of arterial wall will be defined as the single point along an approximate one-centimeter length in which the lumen-intima and media-adventitia interfaces are separated by the greatest distance. Several maximum IMT measurements will be averaged to produce the mean-max IMT. The mean IMT measurement will be defined as the mean-mean of multiple single point IMT measurements across an approximate one centimeter segment of arterial wall. All IMT measurements will be made during end-diastole. One arterial wall segment will be imaged, this will be the common carotid 1 cm proximal to the dilatation of the bifurcation. During each ultrasound examination, the right and left common carotid arteries will be visualized and ultrasound images recorded at a single fixed interrogation angle. The head will be rotated to 45 degrees from the midpoint and the carotid artery scanned in the ear-ear plain. Optimal images will be triggered on the R-wave of the ECG and recorded in DICOM format as still images or as a cine loop onto CD for later offline analysis in the core laboratory. Arterial diameter measurements will also be recorded in order to calculate carotid distensibility.

FMD will be measured using high resolution ultrasound to image the brachial artery in the mid upper arm, proximal to a blood pressure cuff placed just below the antecubital fossa. During each ultrasound examination, the brachial artery will be scanned in longitudinal section and digitized ECG-gated end-diastolic images will be acquired. A hard copy of the B-mode brachial artery image will be stored. Diameter will be determined using an automatic edge-detection algorithm and blood flow measured from the velocity-time integral of the Doppler signal. Reactive hyperemia will be stimulated by inflation of a blood pressure cuff to suprasystolic pressure for 5 minutes, followed by deflation.

The EndoPAT system (endothelium pulse amplitude tonometry) will be used to give a measure of Reactive Hyperemic Index (RHI), a measure of endothelial function designed to be used in conjunction with FMD measurement of reactive hyperemia. The EndoPAT probe is a finger plethysmograph. One probe, formed as a thimble, is placed on each index finger. This probe causes a harmless occlusion of the finger and gives a uniform, near-diastolic external pressure over the distal index finger. The probes will be inflated for five minutes to record a baseline amplitude before the FMD cuff occlusion of the forearm is achieved. The FMD cuff will be inflated for five minutes and then will be deflated. During this time the probes on the index fingers remain inflated and will continue to remain inflated for a further five minutes after cuff deflation. RHI is calculated by integral software.

PWV will be calculated, using measurements obtained by the SphygmoCor system, from pulse pressure waveforms obtained from the carotid, radial and femoral arteries. A pressure tonometer will be placed at the site of each artery for a few seconds until a pressure waveform is recorded and the distance between the carotid-radial and carotid-femoral pulse points will be measured. Integral software calculates the pulse time delay between the points and uses this to give a PWV measurement. All sonographers participating in the cIMT measures will undertake specific training directed by the core laboratory in London (UK).

### Retinal assessment

Annual screening for retinopathy is routinely performed in adolescents with T1D [46]. Anonymised digital copies of the most recent retinal photographs will be collected from each patient at the baseline visit and then during each annual visit until the final assessment. These digital photographs will be used for centralized grading of diabetic retinopathy and, where possible, also for detailed study of the retinal microvasculature.

Stereoscopic fundal photography of seven standard fields is the most sensitive detection methods, which is generally recommended and performed [85]. However, different centers may adopt other validated methods and for the present study, all annually performed retinal photographs with or without mydriasis will be considered. Minimum requirements are a pupil size after dark adaptation of at least 4 mm and two 45-degree photographs taken of each eye, a total of four photographs for retinopathy grading (centered on optic disc and on macula).

Diabetic retinopathy will be graded centrally accordingly to the Early Treatment Diabetic Retinopathy (ETDRS) adaptation of the modified Airlie House classification of diabetic retinopathy [86]. Retinopathy levels for each eye will be classified as follow: level 10: no retinopathy; level 21: at least one microaneurysm or hemorrhage; level 31: microaneurysm plus one or more hemorrhage, exudate, venous bead or loop; level 41: moderately severe non-proliferative retinopathy. The level of retinopathy of the more severely affected eye will be used for assigning the ETDRS grade to a participant.

Digital fundus images should have enough resolution to permit assessment of minor vessel contour. A resolution of 6 microns/pixel or smaller is appropriate. Therefore, where retinal photographs with an adequate resolution and format are available, subclinical retinal microvascular changes, including arteriolar and venular caliber changes, altered vascular fractal dimension, branching and tortuosity, will also be assessed centrally. Retinal vascular caliber and the other vascular aspects will be assessed using a computer program following previously validated protocols [87,88].

### Quality of Life and Health Economics Assessments

An economic evaluation will be conducted to estimate the costs, effects and cost-effectiveness of each IMP and combination therapy in comparison with placebo. It will consist of a within-trial evaluation and a long-term cost-effectiveness analysis using an extrapolation model to estimate costs and outcomes beyond the end of the trial period. Resource use data will be collected on the intervention drugs and consultations and on health care use associated with side effects and complications. Appropriate information will be collected using the CRFs, coupled with a questionnaire *(see additional file *3), administered annually throughout the study period to all 500 randomized and 400 observational subjects, asking about out-of-pocket costs, general practice, out-patients consultations and other expenses and services used. The euroQoI EQ-5D quality of life instrument *(see additional file *4) will also be administered annually; it is widely used and accepted in economic evaluation and permits calculations of utility-based quality adjusted life years. Unit costs will be collected from study centers and from national sources, and will be combined with resource volumes to produce an estimated cost per patient over the study period. Missing data will be dealt with using multiple imputation techniques. Uncertainty will be handled using standard statistical methods and the non-parametric bootstrap.

For the within-trial estimates of cost-effectiveness, effectiveness will be measured using the primary endpoint urinary albumin excretion. For the extrapolated cost-effectiveness, we propose to use the most recent version of the DCCT cost-effectiveness model [89]. This model has been used extensively in economic evaluations of T1D [90] and is available for use by other researchers. The nephropathy module of this model contains a set of nephropathy health states into which the trial's urinary albumin excretion measures can readily be mapped. This will permit long-term cardiovascular morbidity and mortality to be estimated and thence cost per (quality adjusted) life year gained. The model will be adapted to incorporate trial-specific risk factors and population characteristics.

### Statistical analysis

Continuous measures will be compared between groups using analysis of covariance, whereas binary outcomes will be compared using logistic regression. These models will involve adjustment for the randomization stratifiers, and for pre-randomization baseline outcomes to improve precision. The primary endpoint was correlated with baseline standardized ACR in the ORPS cohort (r = 0.3) indicating potential improvement in precision of estimated treatment effects. An initial test will be undertaken for exclusion of the factorial interaction of ACEI with Statin, with subsequent estimation of the factorial main effects of ACEI and Statin and single-arm comparisons. Subgroup analyses, for gender and for baseline standardized ACR (using a median split), will be undertaken by adding an interaction between the subgroup variable and factorial main effects followed by estimation within subgroups. Sensitivity analyses, such as for compliance and for raised HbA1c, will involve analysis using the same methods but applied omitting the relevant subjects or the relevant observations within subjects. For the HbA1c sensitivity analysis exclusions will be defined as those observations following the initial occurrence of a change in HbA1c from baseline in excess of 1%. An analysis restricted to the initial two measurements (first year) where short-term compliance is considered greater, will be performed and contrasted with the later period using within-patient analysis. Missing data will be primarily handled by excluding missing observations from analysis and secondly by multiple imputations [91] considering a contrast of both optimistic and pessimistic scenarios for treatment effects. Analyses will be on the primary basis of intention to treat, with as treated and per protocol as secondary. All tests will be two-tailed, and 5% and 1% significance levels will be used for primary and secondary outcomes respectively.

Formal interim analyses have not been incorporated into the design and analysis of the trial because the 3 to 4 year period of follow-up per subject is long relative to the recruitment period, and an interim analysis would detect early improvements without informing maintenance of any effect. The independent DMEC will determine how regularly to review efficacy and safety regularly, based on progressively longer mean treatment durations.

The sample size was based on consideration of statistical power to detect a worthwhile and plausible treatment effect. There is adequate power to detect main effects in the factorial design and single-arm comparisons. It is thought unlikely that a substantive interaction will exist between ACEI and Statin as mechanisms differ, and the estimation of the size of any interaction effect is not the primary target of the trial. A factorial design is therefore appropriate on grounds of no substantive interaction [92] and interaction not being the primary target [93].

If an interaction, chance or otherwise, should arise then the trial has the safeguard of the single-arm comparisons. In the event of closure of one arm of the trial there would remain adequate statistical power in the single-arm comparisons. A more detailed statistical analysis plan will be developed from the protocol, incorporating guidelines for factorial trials [92,93].

### Adverse Events and Safety Reporting

Adverse event reporting, including suspected unexpected serious adverse reactions (SUSARs), will be carried out in accordance with applicable local regulations and study-specific standard operating procedures. All sites involved in the study will inform the Coordinating center (Cambridge, UK) of any serious adverse events within 24 hours so that appropriate safety reporting procedures can be followed by the Sponsor. All adverse events judged by either the investigator or the sponsor as having a reasonable suspected causal relationship to ACEI or statins therapy qualify as adverse reactions. SUSARs will be reported according to the relevant timelines; expected side effects will be reported in the annual safety report unless serious enough to warrant expedited reporting.

Regular notification of adverse events will be made to the DMEC, principal investigators, the Sponsor, Pfizer and the regulatory authorities (UK Medicines and Healthcare products Regulatory Agency (MHRA), Therapeutic Products Directorate (TPD) of Health Canada, Australian Therapeutic Goods Administration (TGA)).

### Ethical and regulatory issues

#### • Informed Consent

The subjects approached in the UK, Canada and Australia have already consented to screening through centralized assessment of ACR in three consecutive early morning urines. All subjects participating in the RCT will sign an assent form agreeing to the implementation of the full intervention protocol involving the use of both active and placebo medicinal products. As subjects will be aged 11-16 years at recruitment, they will be unable to provide informed consent; this will be obtained from parents or legal guardians according to current ICH, Good Clinical Practice and Medical Research Council guidelines. Both children and the parents will be provided with full information about the trial and adequate time to consider the risk/benefits of participation in the study. On achieving age of consent, the children will be asked to sign forms agreeing to their continued involvement in the study.

#### • Ethical committee review

The study protocol has been approved by the appropriate ethical review committees in the UK, Australia and Canada. The study is to be carried out in accordance with the spirit and the letter of the Declaration of Helsinki and the ICH Good Clinical Practice Guidelines.

#### • Procedures for safety monitoring during the trial

A steering committee has been established to oversee the conduct of the study; a data monitoring and ethics committee (DMEC) will scrutinize the conduct of the study and review data arising from the study in order to determine early stopping rules. SOPs for monitoring adverse events and reactions are in place.

#### • Subject withdrawal

A subject may terminate participation in the study at any time without necessarily giving a reason and without any personal disadvantage. An investigator can stop the participation of a subject after consideration of the benefit/risk ratio. Possible reasons are: 1) serious adverse events; 2) non-compliance; 3) technical grounds (e.g. patient moves away); 4) early termination of the study at the request of the steering committee;

5) pregnancy. The reasons for withdrawal will be clearly documented in the CRF. For subjects who have withdrawn from the study for any of the reasons identified above, permission will be sought to use their data in an 'intention to treat' analysis and for further analyses of long-term outcome data. If they consent to further communication, long-term data collection relating to outcomes will be undertaken.

**Figure 2:**
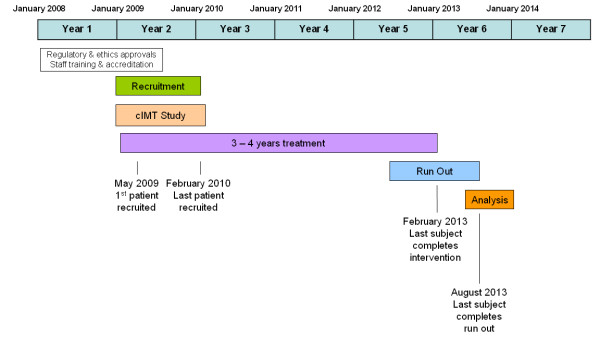
**Timeline and milestones**.

**Study timeline (figure **2**)**

## Discussion

Adolescence is a critical period for the development of vascular complications associated with diabetes [94]. Pubertal development is characterized by many physiological changes, involving both hormonal and metabolic processes, and these factors together with psychological issues are frequently responsible for poor glycaemic control [94]. Treatment may be complicated by poor compliance, difficulties in targeting insulin therapy and concerns about weight gain [94].

It is during puberty that the first signs of both microvascular and macrovascular complications become evident. MA often develops during this period of life [13-17] and represents an independent risk factor for the development of nephropathy and for CVD [12,13]. The development of MA during puberty is often associated with elevated BP, dyslipidemia, decline in renal function, sub-clinical inflammation, endothelial dysfunction and evidence of early atherosclerosis, such as increased cIMT [20-23,25,26,29-32]. These data suggest that, in addition to insulin therapy, intervention directed to provide cardio-renal protection during this critical period may bring long-term benefits in terms of morbidity and mortality. However, although screening for MA during adolescence is strongly recommended [46,95], there is no general consensus as to how MA should be managed.

In adults with persistent MA, ACEI and Statins are increasingly used based on clear evidence of their beneficial cardiovascular and renal effects [56,96]. However, in order to determine whether these agents are also of value in the adolescent population, a clinical RCT is needed.

AdDIT aims to evaluate the efficacy of ACEI and Statins in adolescents with T1D at high risk for DN and CVD, as defined by increased albumin excretion, with the major endpoint of the study being changes in albumin excretion and secondary endpoints including markers of CVD, renal function, retinopathy and early retinal microvascular changes, as well as quality of life combined with detailed assessment of compliance and likely health economic benefits.

AdDIT will provide important data on the potential renal and cardiovascular protective effects of ACEI and statins in high-risk adolescents. As well as determining treatment effect on early surrogate measures of DN and CVD, the trial will incorporate long-term follow-up of the randomized subjects to provide direct evidence of disease outcomes. Follow-up of non-randomized low-risk subjects will determine the potential impact of intervention on long-term risk for DN and CVD. In addition, this study will provide valuable data on tolerance and safety of this therapy as well as data on compliance and health economics.

## Competing interests

The author declares that they have no competing interests.

## Authors' contributions

DBD is acting as the corresponding author for this study.

The AdDIT study Writing Group (M. Loredana Marcovecchio, David B. Dunger, R. Neil Dalton, Denis Daneman, John E. Deanfield, Alastair Gray, Timothy W. Jones, Andrew Neil, A. Toby Prevost) assumes responsibility for the overall content and integrity of this manuscript. All members of the Adolescent type 1 Diabetes cardio-renal Intervention Trial Research Group are listed below. All authors have read and approved the final manuscript.

The AdDIT study Principal Investigators, in addition to those named in the Writing Group above, are: Carlo L Acerini, Rakesh Amin, Binu Anand, Timothy G Barrett, Timothy J Bradley, Fergus J Cameron, David S Celermajer, Timothy D Cheetham, Christopher Cooper, Jennifer Couper, Andrew M Cotterill, Elizabeth A Davis, Kim C Donaghue, Atanu Dutta, Julie A Edge, Nicholas P Mann, Christopher Moudiotis, Gerry Rayman, Julian Shield, Nandu Thalange, Tien Y Wong.

The study coordinators and administrators who have assisted in the set-up and management of the study and development of the protocol are Stella K Silvester, Diane Picton, Yesmino Elia, Lara Moltoni, Barbara Sheil, Barry Widmer, Mark A Wilson, Tracey J Stevens, Adelyn L Thomason, Ravneet Phalora, Sarah Kok.

## Pre-publication history

The pre-publication history for this paper can be accessed here:

http://www.biomedcentral.com/1471-2431/9/79/prepub

## Supplementary Material

Additional file 1 **AdDIT study centers**. List of the centers world-wide participating in AdDIT, with numbers of potential participants in each center. Click here for file

Additional file 2 **Adverse reactions to the study drugs**. Expected side effects of the study drugs, Atorvastatin and Quinapril, and how they will be managed including pregnancy and in utero exposure. Click here for file

Additional file 3 **Health Economic Assessment**. Questionnaire to be completed by participants/their parents describing resource usage of health and social services over the last year. Click here for file

Additional file 4 **Quality of Life Questionnaire**. EQ-5D health questionnaire and health scale, to be completed by participants to assess quality of life. Click here for file
