# Supplementary material for: Adolescent type 1 Diabetes cardio-renal Intervention Trial (AdDIT)
Source: BMC Pediatr. 2009 Dec 17;9:79. doi: 10.1186/1471-2431-9-79 (PMC2814806; doi:10.1186/1471-2431-9-79)
Supplement: Additional file 1 — AdDIT study centers. List of the centers world-wide participating in AdDIT, with numbers of potential participants in each center. [file 1471-2431-9-79-S1.DOC]

## Additional file 1

| **AdDIT study centers** | | **Potential participants**  **(age 11-16 y)** |
| --- | --- | --- |
| **East Anglia, UK** | 1. Addenbrooke's Hospital, Cambridge  2. Hinchingbrooke Hospital, Huntingdon  3. Peterborough General Hospital  4. Norfolk & Norwich Hospital, Norwich  5. West Suffolk Hospital, Bury St. Edmunds  6. Northampton Hospital  7. Ipswich Hospital | 150  50  50  50  50  50  50 |
| **Oxfordshire, UK** | 1. John Radcliffe Hospital, Oxford  2. Royal Berkshire Hospital, Reading  3. Stoke Mandeville Hospital, Aylesbury | 160  150  100 |
| **South West, UK** | 1. Bristol Royal Hospital for Sick Children, Bristol  2. Southmead Hospital, North Bristol  3. Weston General Hospital, Weston-Super-Mare | 200  150  100 |
| **Birmingham, UK** | 1. Birmingham Children's Hospital  2. Birmingham Heartlands Hospital | 250  100 |
| **North West, UK** | 1. Royal Manchester Children's Hospital, Manchester  2. Stepping Hill Hospital, Stockport | 250  100 |
| **Newcastle, UK** | Royal Victoria Infirmary, Newcastle Upon Tyne | 300 |
| **Western Australia** | Princess Margaret Hospital for Children, Perth | 280 |
| **New South Wales, Australia** | Children’s Hospital Westmead, Sydney | 530 |
| **South Australia** | Women’s and Children’s Hospital, North Adelaide | 280 |
| **Victoria, Australia** | Royal Children’s Hospital, Melbourne | 580 |
| **Queensland, Australia** | Mater Children’s Hospital, Brisbane | 200 |
| **Toronto, Canada** | Hospital for Sick Children (SickKids), Toronto | 350 |
| **Other sites in Ontario, Canada** | | 150 |
