# Supplementary material for: Adolescent type 1 Diabetes cardio-renal Intervention Trial (AdDIT)
Source: BMC Pediatr. 2009 Dec 17;9:79. doi: 10.1186/1471-2431-9-79 (PMC2814806; doi:10.1186/1471-2431-9-79)
Supplement: Additional file 2 — Adverse reactions to the study drugs. Expected side effects of the study drugs, Atorvastatin and Quinapril, and how they will be managed including pregnancy and in utero exposure. [file 1471-2431-9-79-S2.DOC]

### Additional file 2

### Attempts to limit the side effects and adverse effects of study drugs

**Quinapril (Study drug Q):**

Cough and hypotension: in order to reduce the occurrence of these complications, study drug Q will be introduced at a low dose of 5mg increasing to 10mg after 2 weeks provided that no adverse events are experienced. If adverse events are present the participant will continue on 5mg and will be reassessed at subsequent trial visits to determine if study drug Q can be increased to 10mg. It is intended that participants will be maintained on a dose 10mg if possible and tolerance closely monitored. If at any time during the study cough and/or hypotension occur, the dose of drug Q will be reduced to 5mg. Investigators will re-evaluate symptoms for signs of improvement at subsequent trial visits and make a decision to re-start or increase study drug Q. Where necessary, study drug Q will be discontinued but the patient will continue to take study drug A and participate in the trial.

Hyperkalemia. As increases in serum potassium have been reported in patients treated with Quinapril, potassium blood levels will be monitored during therapy. Hyperkalemia is defined by potassium levels > 6 mmol/L. Patients will be told not to use potassium supplements, and/or potassium containing salt substitutes, as they increase the risk of hyperkalemia. If hyperkalemia develops, the dose will be reduced or, where necessary, the drug will be discontinued but the patient will continue to participate in the trial

GFR decline. GFR will be assessed at the beginning of the study, one month after first taking the study drugs and then every six months. A decline in GFR of 25% or greater, as assessed by a creatinine-based formula (eGFR (ml/min/1.73m^2^) = 42*height (cm)/plasma creatinine (μmol/l), will indicate a significant impairment of renal function and this will result in withdrawal from the study drug Q but the patient will continue to participate in the trial.

**Atorvastatin (Study drug A):**

Alteration in liver function tests: ALT levels will be checked at baseline, one month after starting treatment and then every six months during the intervention period. If at any time during the study, ALT levels exceed more than 3 times the normal upper limit, the patient will be scheduled for a repeat ALT evaluation within 1 week (±3 days). If the repeat value is still 3 times above the normal range the patient will have the double-blind study drug A discontinued but will continue to participate in the trial.

Muscle pain and Rhabdomyolysis: All subjects will be advised to report promptly unexplained muscle pain, tenderness or weakness, especially if accompanied by malaise or fever. In the case of myalgia symptoms, blood will be drawn for CK levels and ALT. The following criteria will be used to assess CK results for possible Myopathy. A CK result 10 times the upper limit of normal with muscle symptoms and a raised ALT of 3 times the normal upper limit will require a SAE report to be completed. Study drug A will be stopped immediately and permanently to avoid renal impairment.

A CK level result 10 times the upper limit of normal with muscle symptoms and with a normal ALT result will be repeated in one week along with the ALT. A CK level result 10 times the upper limit of normal in a subject that then becomes asymptomatic will be re-checked within one week along with ALT. If the increased CK levels are not associated to muscle pain the investigator will evaluate whether or not it is necessary to stop the study drug. A CK result 5 times or greater the upper limit of normal irrespective of symptoms will be re-checked within one week along with ALT.

**Quinapril and Atorvastatin**

Exposure in Utero and Pregnancy associated complications

Both Quinapril and Atorvastatin are contra-indicated during pregnancy because of concern about teratogenecity and because they may interfere with early fetal growth and result in stillbirth.

Exposure In Utero

For investigational products and for marketed products, an exposure in-utero (EIU) occurs if:

1) a female becomes, or is found to be, pregnant either while receiving or having been directly exposed to (e.g., environmental exposure) the investigational product, or the female becomes, or is found to be, pregnant after discontinuing and/or being directly exposed to the investigational product (maternal exposure);

2) a male has been exposed, either due to treatment or environmental, to the investigational product prior to or around the time of conception and/or is exposed during his partner’s pregnancy (paternal exposure).

If any study subject or study subject’s partner becomes or is found to be pregnant during the study subject’s treatment with the investigational product, the investigator must submit this information to Pfizer on an Exposure in Utero Form, and to the Regulatory Authorities. In addition, the investigator must submit information regarding environmental exposure to a Pfizer product in a pregnant woman (e.g., a nurse reports that she is pregnant and has been exposed to a cytotoxic product by inhalation or spillage) using the Exposure in Utero Form. This must be done irrespective of whether an adverse event has occurred and within 24 hours of awareness of the pregnancy. The information submitted should include the anticipated date of delivery (see below for information related to induced termination of pregnancy).

Follow-up is conducted to obtain pregnancy outcome information on all Exposure in Utero reports with an unknown outcome. The investigator will follow the pregnancy until completion or until pregnancy termination (i.e., induced abortion) and then notify Pfizer and the Regulatory Authorities of the outcome. The investigator will provide this information as a follow up to the initial Exposure in Utero Form. The reason(s) for an induced abortion should be specified. An EIU report is not created when an ectopic pregnancy report is received since this pregnancy is not usually viable. Rather, a serious adverse event case is created with the event of ectopic pregnancy.

If the outcome of the pregnancy meets the criteria for immediate classification as a serious adverse event (i.e., spontaneous abortion, stillbirth, neonatal death, or congenital anomaly [including that in an aborted fetus, stillbirth or neonatal death]), the investigator should follow the procedures for reporting serious adverse events.

In the case of a live birth, the “normality” of the newborn can be assessed at the time of birth (i.e., no minimum follow-up period of a presumably normal infant is required before an Exposure in Utero Form can be completed). The “normality” of an aborted fetus can be assessed by gross visual inspection, unless pre-abortion test findings are suggestive of a congenital anomaly.

Additional information about pregnancy outcomes that are classified as serious adverse events follows:

- “Spontaneous abortion” includes miscarriage and missed abortion.
- “All neonatal deaths that occur within 1 month of birth should be reported, without regard to causality, as serious adverse events. In addition, any infant death after 1 month that the investigator assesses as possibly related to the exposure in utero to the study drugs should be reported.

Additional information regarding the exposure in utero may be requested by the investigator. Further follow-up of birth outcomes will be handled on a case-by-case basis (e.g., follow-up on preterm infants to identify developmental delays).In the case of paternal exposure, the investigator must obtain permission from the subject’s partner in order to conduct any follow-up or collect any information.

Post-menarchal female subjects and their parents will be informed of the potential risks and the need for trial patients to use contraception if sexually active. They will be asked to provide urine samples for 6-monthly pregnancy tests. SOPs will be put in place to deal with any pregnancy, including immediate unblinding and comprehensive independent counseling as to the advisability of continuing the pregnancy.
