# Supplementary material for: Adolescent type 1 Diabetes cardio-renal Intervention Trial (AdDIT)
Source: BMC Pediatr. 2009 Dec 17;9:79. doi: 10.1186/1471-2431-9-79 (PMC2814806; doi:10.1186/1471-2431-9-79)
Supplement: Additional file 3 — Health Economic Assessment. Questionnaire to be completed by participants/their parents describing resource usage of health and social services over the last year. [file 1471-2431-9-79-S3.DOC]

Additional file 3

Resource usage (postal questionnaire to patient)

We would like to know how much use you have made of the health and social services over the last 12 months. If you are not exactly sure, we would rather have your best guess than no information at all. Please answer every question, even if the answer is "0".

0

3

Please fill in both boxes, for example: if seen three times.

Over the last 12 months, how many times have you:

| 1. | Been seen by your GP ?.................................................. |  |  |
| --- | --- | --- | --- |
|  |  |  |  |
| 2. | Been seen by a practice nurse ?...................................... |  |  |
|  |  |  |  |
| 3. | Been seen by a health visitor ? |  |  |
|  |  |  |  |
| 4. | Been seen by an optician or visited an eye clinic?............. |  |  |
|  |  |  |  |
| 5. | Visited a diabetes clinic?.................................................. |  |  |
|  |  |  |  |
| 6. | Visited any other hospital clinic?...................................... |  |  |
|  |  |  |  |
| 7. | Visited a day hospital?...................................................... |  |  |
|  |  |  |  |
| 8. | Been in hospital at least overnight?.................................. |  |  |
|  |  |  |  |
|  | If you have been in hospital, please enter the total  number of all nights in hospital here |  |  |

Over the last year, roughly how much have you or your family and friends had to pay
directly for anything such as equipment, supplies, medications, or travelling costs related to your diabetes. (An approximate figure will be fine.) £

If you would like to make any other comments, please write them here.

……………………………………………………………………………………………

**Many thanks for your help**
