# Supplementary material for: Adolescent type 1 Diabetes cardio-renal Intervention Trial (AdDIT)
Source: BMC Pediatr. 2009 Dec 17;9:79. doi: 10.1186/1471-2431-9-79 (PMC2814806; doi:10.1186/1471-2431-9-79)
Supplement: Additional file 4 — Quality of Life Questionnaire. EQ-5D health questionnaire and health scale, to be completed by participants to assess quality of life. [file 1471-2431-9-79-S4.DOC]

Additional file 4: Quality of Life Questionnaires

EQ-5D Health Questionnaire

To help people say how good or bad a health state is, we have drawn a scale (rather like a thermometer) on which the best state you can imagine is marked 100 and the worst state you can imagine is marked 0.

We would like you to indicate on this scale how good or bad your own health is today, in your opinion. Please do this by drawing a line from the box below to whichever point on the scale indicates how good or bad your health state is today.
